# Current Advances of Artificial Intelligence and Machine Learning in Orthopaedics: A Focus on Hip Surgery

**DOI:** 10.3390/bioengineering12121353

**Published:** 2025-12-11

**Authors:** Alberto Di Martino, Chiara Di Censo, Enrico Masi, Manuele Morandi Guaitoli, Giuseppe Geraci, Cesare Faldini

**Affiliations:** 1 First Orthopaedic and Traumatologic Department, IRCCS Istituto Ortopedico Rizzoli, via Giulio Cesare Pupilli 1, 40136 Bologna, Italy; chiara.dicenso@ior.it (C.D.C.);; 2 Department of Biomedical and Neuromotor Science-DIBINEM, University of Bologna, 40136 Bologna, Italy

**Keywords:** artificial intelligence, hip, diagnosis, total hip arthroplasty, radiology

## Abstract

In recent years, we assisted the exploitation of Artificial Intelligence (AI) that invasively pervades in several instances of everyday life. The potential of this technology promises the automation of human tasks increasing accuracy and efficiency. The integration of AI systems in the orthopaedic field is becoming more and more a concrete reality, so this topic is gaining increasing interest by the scientific community. More and more authors are testing the power of AI in orthopaedics, exploiting the application in routine workflow, and asking if AI could improve clinical and surgical practice. In this brief narrative review, the state-of-art of AI in hip district orthopaedics is presented, particularly focusing on the application of AI tools in the context of radiological images, early diagnosis, clinical datasets, and around operative theatre. Possible future development of AI-hip pathology management is exposed too, and clear doubts about exploits of these tools in clinical practice are also exposed.

## 1. Introduction

A proliferation of Artificial Intelligence (AI) applications in several scientific and technological domains, alongside the increasing availability and utilisation by the general internet user, has been observed in recent years. Simultaneously, study of potential applications in medical and surgical fields emerged, and the literature on the use of AI to support clinical practice is on the rise [1].

AI presents numerous potential applications within the field of orthopaedics, alongside a comparable number of prospective future advancements. In prior years, the utilisation of AI in orthopaedics was primarily focused on cost predictions within the insurance sector [2,3] and automated reporting of musculoskeletal imaging [4]. Currently, however, the scope of AI application appears considerably broader, encompassing patient education [5,6], diagnosis assistance, preoperative planning, design of custom devices, and intraoperative execution with the adoption of physical AI (robotic surgery) [7,8], to meticulous postoperative follow-up and rehabilitation considering also the possible prediction of clinical outcomes.

Nevertheless, AI remains a highly innovative technology, relatively unexplored by most orthopaedic surgeons practicing outside of academic settings. Its progression is significantly more rapid than the ability of surgeons to stay informed and leverage it appropriately and conscientiously. Notably, the challenges that classically plague the naive and premature adoption of AI are also emerging in orthopaedics: “alignment” problems and ethical concerns, as well as potential medicolegal implications, may, in certain contexts, substantially moderate the potential benefits of this technology.

The purpose of the current review is to outline how AI is currently applied in the orthopaedic setting. As the application of AI in medical and orthopaedic fields is broad, the referred review will focus on hip pathology and surgery, highlighting recent advancements, exploring the multifaceted benefits it offers, and addressing the inherent challenges and future directions that lie ahead in this dynamic and rapidly evolving field.

To the best of the authors’ knowledge, no prior comprehensive review specifically addressing the application of AI within the context of hip surgery has been conducted.

## 2. AI Systems

Most medical users might be unfamiliar with definitions, types, and current applications of this technology; thus, it is necessary to draft a brief and concise description of AI and Machine Learning technologies. AI is a broad term encompassing several fields within computer science. For this reason, it is challenging to arrive at a single, definitive definition [9]. AI can be considered a general term that encloses Machine Learning (ML), and Deep Learning (DL). The AI-based learning systems, as ML and DL, are being increasingly applied in medicine, and are being revealed as support tools for several areas, for instance, instrumental diagnostics, but also preoperative planning, clinical decision-making, or dataset analysis. The expected goal of an AI tool is to support humans to solve complex problems, choosing the best possible action to complete a given task.

From the first ancestors of algorithms that date back to 1950, during the Second World War, AI has been becoming an emerging and growing technology, with increasing popularity thanks to the spread of this technology to public users.

AI systems have been applied in several fields, and health AI-based programmes have been developed. Even if AI represents a powerful and advanced tool, its definition is hard to outline, in relation to how the term “intelligence” is meant to be defined [10]. To simplify the matter, AI is related to a computer machine that, given a definitive environment and a target goal, is able to choose the best action to succeed the task. Alongside this, it might be useful to become familiar with the definition given by the European Commission’s High-Level Expert Group On Artificial Intelligence: “Artificial intelligence (AI) refers to systems that display intelligent behaviour by analysing their environment and taking actions—with some degree of autonomy—to achieve specific goals.” [11].

AI systems provide support to humans to perform complex tasks, and the application of this tool in daily occupations is growing rapidly. The AI innovation regards performing actions that are simple and immediate for human beings, but extremely complex for a machine. In fact, humans find it extremely easy to recognise an animal in a set of objects, but they suffer and have difficulty with the execution of long calculations. As a contrary, a computer machine is able to rapidly manage big numbers, but it does not easily recognize a face. These tasks are commonly associated with complex cognitive functions that are typical of the human mind, such as learning, planning, or organizing.

Machine Learning (ML) can be simply described as a scientific field that studies how computers can learn from data. The ML building process is performed with several learning techniques such as Supervised Learning, Unsupervised Learning, and Reinforcement Learning (Table 1). Learning approaches allow uses and applications of AI and the performance of different tasks that are discussed below [12].

In Supervised Learning, the learner is trained with both labelled input and output data at first, i.e., a set of data in which input items are clearly connected to the outputs, so that the learner can detect the best association between items. As the training phase is completed, the learner is able to infer with the right outcome, given a known input, even with items unseen before [13].

This kind of technology best fits in retrospective studies in which input and outcomes are well defined. As the inlet and outlet data are known, a supervised ML model learns the best connection between data, and once trained, these could predict probable events in new patients. Mohammadi et al. [14], for instance, applied a supervised ML model to find patients who underwent THA or TKA at risk for unplanned readmissions. The ultimate goal was developing an AI/ML algorithm able to predict the risk of 30-days readmission on the basis of clinical reports on Electronic Hospital Records (EHRs). The authors found a satisfactory discriminative ability of the ML tool tested, even if additional validation and empirical evidence are necessary to be integrated as clinical support [15].

On the other hand, in Unsupervised Learning, training data are not linked and there is not a defined x, y association. Unsupervised Learning runs datasets with the aim to group, analyse and evaluate raw items. For example, several images are presented without labelling if an animal is represented or not; the learner would suggest the best possible classification by the analysis of the available dataset. It is important to underline that this technique is able to highlight unconventional clustering of data which could be grouped for features that could not clearly be evident to humans [9].

Finally, Reinforcement Learning is an approach based on the action–reward mechanism: the learner is free to perform all the possible actions, receiving a reward for the right ones. In this way, the strongest connections are preferred above the weak ones; therefore, the learner would run the positively reinforced tasks over time [16].

The three approaches require human intervention in data presentation to transform raw data into items that are feasible for the ML. This process requires precise expertise on data management, and it is an extremely time-consuming process. For these reasons, these approaches are also known as “Conventional Learning”, to distinguish it from Deep Learning (DL) [17], which is an advanced method of learning that does not require manipulation of data (Table 2). DL models are capable of “learning” from data, in the sense that they can analyse raw data and extract the required features automatically. This process, however, can be performed if the DL model is fed with large datasets, to build a proper data-driven model. Indeed, differently from conventional ML, DL models offer poor performance if built with a small dataset.

**Figure 1:**
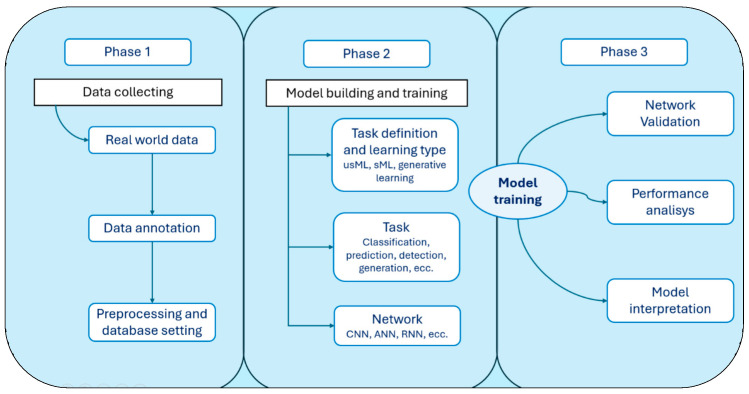
Schematic representation of the data collection and processing process of a Machine Learning system. The presented data can be processed in different ways depending on the outcome you want to obtain from the machine. CNN, Convolutional Neural Network; RNN, Recurrent Neural Network, ANN, Artificial Neural Network, sML, Supervised Machine Learning, usML, Unsupervised Machine Learning.

The DL building process can be divided into three phases (Figure 1): a first data preparation phase, in which data are collected from real-world sources, and their understanding and pre-processing steps are performed; then, a DL model is built and trained with the collected data, the learning technique is set, and the task is defined; finally, the model is defined and validated [17]. These learning techniques have progressively found more applications in the field of healthcare, and the uses in orthopaedics are discussed below.

## 3. Materials and Method

**Figure 2:**
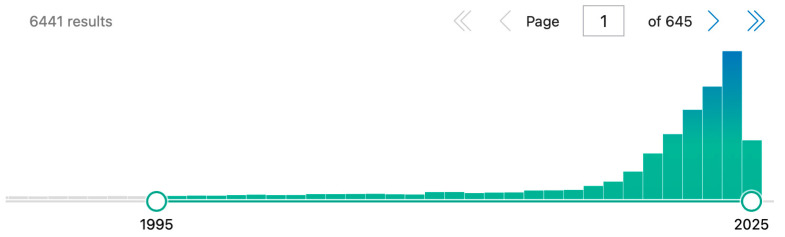
PubMed results searching the strings “((artificial intelligence) OR (machine learning)) AND ((orthopaedic) OR (orthopaedic))”, proving the relevance in the literature of this argument over the last 30 years (date of access 10 April 2025).

The narrative review has been conducted using the PubMed research tool. As “artificial intelligence”, “orthopaedic”, and “hip” represent the current topic, the research has been divided as follows: (1) a broad research has been conducted as mentioned in Figure 2 including all published items and without time restriction to evaluate the extent of AI application in orthopaedic studies in the literature up to April 2025; (2) relevant subtopics in orthopaedics with AI application have been selected, and include Diagnostic Imaging, traumatology and hip fractures, preoperative total hip arthroplasty planning, postoperative total hip arthroplasty complications. Only published studies have been selected. The subtopic research has been restricted to randomized control trials, clinical studies, comparative studies, and systematic reviews. No time restriction has been applied in either of the two phases.

### 3.1. Diagnostic Imaging

One of the first fields in which AI matched with orthopaedics is Diagnostic Imaging. While the applications in clinical practice are still under study and far from systematic employment, radiology is the ambit in which AI has found major developments, thanks to certain characteristics of the discipline that are particularly well suited to the field of AI, including the enormous amount of data in digital format gathered from Institutions’ PACS (Picture Archive and Communicating Systems) and the possibility to run collected data by ML software. At present, radiology represents the first domain per number of Machine Learning-enabled Medical Devices, according to the Food and Drug Administration (FDA) [18].

The radiological image dataset can be analysed by AI tools which aid the specialist in the interpretation of images and the detection of incidental findings which could be hidden to the human eye, and automatic anatomical measurements [19]. AI systems, in addition to supporting image interpretation in radiology, could also be used to automate the generation of reports, using the integration of Machine Learning and Deep Learning systems with Natural Language Models (NLS) [20].

**Figure 3:**
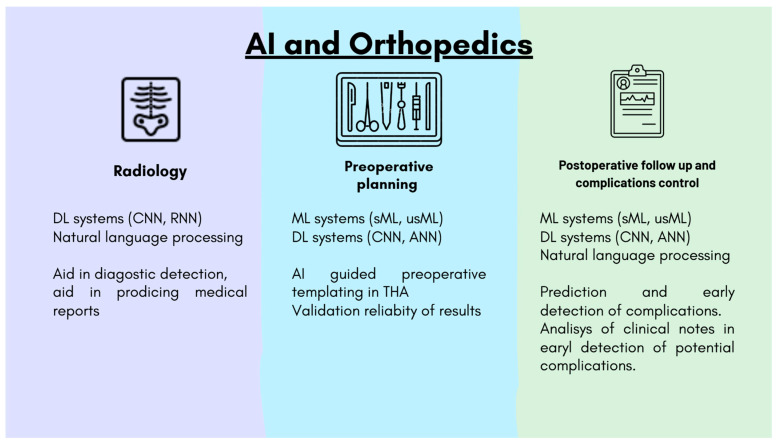
Potential application of AI tools in orthopaedic fields.

The application of ML systems has been assessed in various medical fields, such as dermatology [21], cardiology, or ophthalmology and compared with health specialists performances, with good results in favour to ML. However, the potentialities of AI in orthopaedics are still growing and under development. Recent body of knowledge about the application of AI tools in radiology has been increasing [22], and many published manuscripts in recent years have outlined the potentials of AI systems in orthopaedic radiology (Figure 3).

ML can run large databases and learn from huge volume of data, representing a technology that is easily applicable in radiology. On the other side, images represent optimal data type for ML systems, that can be processed for Deep Analysis. ML algorithms have been tested to recognize pathological patterns and to classify pathological images through diagnostic criteria [23]. Xue et al. trained, validated, and tested a Deep Learning-based algorithm in the identification of osteoarthritis in standard pelvic radiography (SPR), comparing the performances with a group of experts. X-rays were labelled as “normal” or “osteoarthritis (OA)”. The authors found high specificity (95.0%), high sensibility (90.2%), and good accuracy of the tested network. Such potentialities of AI could be used in the early identification of pathological processes, which are difficult to identify by standard X-rays and are easily misdiagnosed, to identify groups of patients at-risk to develop the disease, that should be followed-up overtime. Shen et al. [24] performed a retrospective study on magnetic resonance images (MRI) of patients diagnosed with early osteonecrosis of femoral head. They compared the ability of the ML model to target the positive images with opinions of a group of surgeons, finding excellent results in terms of sensitivity, specificity, and accuracy of the ML model.

Another determinant issue in the implementation of AI systems in Diagnostic Imaging also lies on the rapidity in data processing, reducing time spent for radiological interpretation, with potential contribution in alleviating daily workload. In an era based on instrumental diagnostics, in which the number of imaging exams per patient is projected to increase, AI technologies could represent a valid solution to reduce the workload for specialists, increasing efficiency and alleviating resource waste [22].

In the following paragraphs, current and potential applications of ML and DL models for diagnostics in orthopaedics will be highlighted, focusing on hip pathology. Specifically, innovations in the diagnostics of primary hip pathology and secondly, AI systems applied to trauma diagnostics will be discussed.

### 3.2. Congenital or Developmental Hip Diseases

AI systems have been tested in recent years for diagnostic support in the assessment of hip developmental and acquired pathologies. Radiographic examinations are still the principal method employed in the assessment of hip pathologies, also applied for diagnosis, surgical treatment, preoperative planning, and postoperative check-up. The diagnostic process for differential diagnosis of hip pathologies is associated with measurements of the main parameters of the hip joint [25]. These measurements are frequently executed manually through conventional X-ray software, leading to relevant problems in terms of reproducibility and being at risk of methodological errors.

In this way, the application of AI to the diagnosis of hip pathologies could be resolutive in decreasing measurements errors, identification of borderline cases, and improving consistency in diagnostic process. Moreover, AI support could be useful in early screening of developmental hip disorders impacting the early selection of patients potentially at risk, as well as leading to targeted, patient-specific treatments.

**Figure 4:**
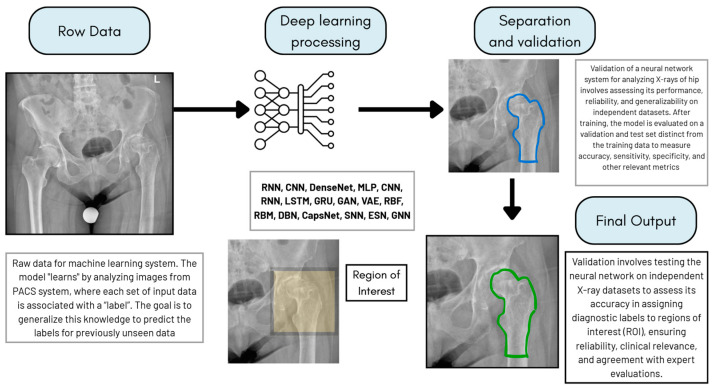
Scheme reporting the ML workflow in Supervised Learning mode.

The potential of AI systems applied to the diagnosis of hip pathology is currently being explored, with a growing body of literature on the subject: AI systems have been tested in the early detection of hip pathological patterns and compared to human performances, they are being demonstrated as a good opportunity for early detection of developmental diseases in orthopaedics such as Developmental Hip Dysplasia (DDH) (Figure 4), Legg-Calvè Perthes disease, Slipped Capital Femoral Epiphysis, or osteonecrosis of femoral head (ONFH).

Xu et al. [26] conducted a clinical study to evaluate the performance of a DL system based on a Deep CNN in the radiographic detection of DDH in children up to 3 years old, comparing machine and surgeon performances. An amount of 1398 X-rays were separately evaluated by AI and by a senior surgeon, an intermediate surgeon, and a junior surgeon. The authors considered radiological indicators for DDH assessment so that the Tonnis classification and International Hip Dysplasia Institute (IHDI) classification could be applied. AI and clinicians were asked to measure indicators like Shenton line, lateral edge of acetabulum, and sourcil of acetabulum bilaterally. Acetabular index and central edge angle have been assessed, and Tonnis grade and IHDI grade were calculated automatically. Accuracy, sensibility, and specificity of the AI system and surgeon at different expertise have been evaluated and compared, demonstrating AI performances match closely to the intermediate surgeon. Time spent to reach the diagnosis has been collected too for both AI and surgeons. The authors did not find a significant difference in these parameters between surgeons and AI systems in the calculated parameters, even if time used to obtain the DDH indicators on X-rays was significantly shorter for AI with respect to the surgeons (*p* < 0.001).

AI-based technologies were developed as a support in operator-based exams. In orthopaedics, ultrasound (US) examination is a crucial tool for the early diagnosis of DDH. However, the exam is negatively influenced by operator’s experience, because a reduced experience is related to errors in probe orientation, with misleading anatomical images and lack of adequacy, which could hamper the final interpretation [27]. Paserin et al. [28] developed an ML system formed by an integration of Convolutional Neural Network–Recurrent Neural Network (CNN-RNN) architecture, able to support the operator automatically during the acquisition of images meaningful to train the CNN-RNN on. The Alpha-angle and Beta-angle were defined and the window required for each scan was determined. The DL model proposed in the study was designed to merge information of interslice images for 3D volume US elaboration and define the adequacy of the volume selected for DDH diagnosis. The architecture allowed the complete scanning of US slices as they were acquired. The tested network correctly classified 14 out of 20 test volumes. This architecture gained an accuracy of 82%, with low processing time (almost 1 s), supporting its use in general practice, being potentially able to strongly impact on the paediatric population screened for DDH.

### 3.3. Avascular Necrosis of the Hip

Avascular Necrosis of the Hip (AVNH) is one of the principal chronic hip pain diseases caused by an interruption of blood supply to the femoral head leading to bone necrosis. The pathological process starts silently, so that at the symptomatic onset, the necrotic process is in advanced phase. Radiography is the principal diagnostic test, but presents low sensitivity in early stages, where MRI is most sensitive instead.

DL could significantly impact the management of this pathology. At first, early signs of AVNH could be detected through standard radiography, supporting clinicians in detecting patients deserving attention. Chee et al. [29] developed a DL algorithm to address signs of AVNH from hip anteroposterior radiographs of patients 16 years old or older, previously identified with this pathology on MRI. Sensitivity assessment was the main goal of the study. The performance of the software was compared with the interpretation of a group of radiologists, finding a non-inferiority of the network examined versus the radiologist examination. As the management of AVNH depends on the pathological stage, and the necrosis progression leads to more invasive surgeries such as THA, the detection of patients in early AVNH is impactful for a conservative and less invasive intervention. MRI could detect the early sign of femoral head necrosis and significantly impact the patient management; however, the MRI-based diagnosis is a time-consuming process and the accuracy is influenced by the specialist expertise. AI enters this field to ease the diagnosis at an early stage, and to help the radiologist for standardized recognition of MRI images. This is particularly useful as early-stage femoral head AVNH which could benefit from non-surgical treatments (e.g., bisphosphonates) or preservative surgery. In the work of Shen et al. [24], a DL algorithm was analysed in the investigation of MRI images collected at an early stage in patients with a definite diagnosis of AVNH, demonstrating that the network was capable of performing similarly to radiologists with experience. At the current state of the art, ML-based algorithms could implement the diagnostic workflow as adjuvant tools for the physician. In contrast to other studies on AVNH AI-based diagnosis, MRI scans provide complex and complete anatomical characteristics, so the DL processing needs to manage more information to obtain adequate performances. The results obtained by the authors in terms of specificity, sensibility, and accuracy are like chief surgeons. These performances are strictly required by such tools in order to open a possible clinical application. However, a possible consideration in the application of AI on early assessment of AVNH is the risk of overdiagnosis related to a blind and irrational application of the system to clinical practice. In this way, overreliance on AI outcomes should be avoided, and the results obtained by the system should be critically evaluated by the clinician [30].

## 4. AI and Hip Fractures

Hip fractures (HF) represent one of the major impacts of public health traumas, due to the increasing global incidence and associated risk of morbidity and mortality. The reported 1 year mortality rate after femoral neck fracture is about 20HF, and commonly occurs in the elderly, especially after the age of 75, and mostly after a fall with impaction over the lateral aspect of the tight. With the progressive aging of the population, HFs are expected to progressively increase; in 1997, Gullberg et al. predicted a doubled incidence of HF by 2050 with a prevalence up to 21.3 million [31]. In Italy, the reported incidence for HF is above 300 for women and 150 for men over 100.000 inhabitants [32]. The relevance of HF resides in the costs associated to their management and treatment, with direct costs associated to HF comparable to the costs of Myocardial Infarction in the same country [33,34,35,36,37].

Conventional radiography is the principal diagnostic method to assess HF, as a low cost and available diagnostic technique. Plain radiographs with anterior posterior view of the pelvis are the standard projection for the diagnostic assessment. Secondary Diagnostic Imaging, as computed tomography or magnetic resonance imaging, is required in case of occult hip fractures [32].

Several studies demonstrated improved outcomes in terms of mortality, pain, complications, and length of stay associated to a quick surgical management [38,39]. Moreover, the mortality rate after one year has been revealed to be significantly lower in patient treated within 48 h with respect to patients treated between 2 and 3 days.

As defined by the relevance of HF, a proper diagnosis and management is imperative. Missed diagnosis could occur and, in some cases, the prevalence has been reported as high as 10% [40]. In this context, AI algorithms could be applied in the assessment of patients with lower limb trauma and in the prediction of outcomes. Several AI-based models have been developed to help clinicians in the diagnosis and classification of HF [41,42,43]. ML tools were built up and tested on standard pelvic radiographs (SPRs), and their performance with selected databases of radiographic images was evaluated in terms of specificity, sensitivity, and accuracy. Cheng et al. [44] tested a Human Algorithm Integration system (HAI) based on a Deep Convolutional Neural Network (DCNN) architecture to detect HF. The system was tested in a real clinical workflow, and performances were analysed. The network was trained with 25,505 SPR and tested with 3605 images, evaluating accuracy, sensitivity, specificity, and rate of false negatives of the model. The authors compared the performance of the model with a group of 34 clinicians with different levels of expertise, and they found good scores in terms of accuracy (91%) and sensitivity (98%) of DCNN. Moreover, the system was applied at three local trauma centres to support HF diagnosis using SPR, expressing the probability of the presence of fractures. Tested images were presented to a clinician that provided the final validation of diagnosis. Highest values in terms of sensibility, sensitivity, and accuracy were reported in the combined performance, compared with human- or algorithm-derived alone.

Gao et al. [45] examined the performance of a DCNN on a total of 40,000 images, using image classifier methodology. The authors obtained high results in sensitivity, specificity, and accuracy (96.1% in accuracy, 94.2% in sensitivity, and 96.3 in specificity), and good negative predictive value. However, the authors claim limitation in comparability of the algorithm with similar models because of the heterogeneous inclusion criteria, and because of the monocentric setting of the study.

AI-based diagnostics represent a promising support tool for the identification of HF, particularly in light of their rising incidence and substantial impact on public healthcare costs. Research in this field is ongoing, and its potential benefits—such as reducing healthcare expenditures and alleviating clinical workload—are increasingly evident. AI algorithms, particularly those based on DL, have demonstrated high accuracy, sensitivity, and specificity in detecting HF on standard radiographs, often outperforming less experienced clinicians. In addition, AI can assist in triaging cases, minimizing diagnostic delays in emergency settings and improving patient outcomes. When integrated into clinical workflows, these tools can enhance diagnostic consistency and reduce the risk of missed fractures, especially in high-volume or resource-limited environments.

## 5. Preoperative Templating of Total Hip Arthroplasty (THA)

THA is the elective treatment for end-stage hip osteoarthritis (OA) when symptoms compromise the patient’s quality of life. It is one the most successful surgical procedures among arthroplasty surgery in terms of implant survival and clinical reversion [46].

The large success of THA can be attributed to improvement in surgical technique and patient selection as well as of the development component materials [47,48]. Main challenges to afford are leg length discrepancy, and the risk of dislocation and implant failure. Preoperative templating for THA aims to select optimal component models, sizes, and the best implant positioning based on patient’s characteristics and anatomical conformation of the hip and femur [49]. Several studies show that unplanned THA are associated with poor results and higher costs than the planned ones [50]. THA templating has been historically performed by two-dimensional (2D) acetate templating, while currently, the 2D digital technique is the standard in institutions from developed countries. It is the most diffused operator-dependent procedure performed by computer software on digital X-ray images; it is an accurate and reproducible method, easy to perform, and cost-effective [51]. Implementing X-rays, 2D planning is subjected to errors derived from the machine magnification and patient positioning, as well as the projection of errors related to X-ray acquisition. Three-dimensional digital planning is usually performed after CT scan acquisition of the pelvis and femurs up to the knees; therefore, it allows a three-planar study of hip anatomy and biomechanics, and a three-dimensional preview of the reciprocal position of implants’ components. The limitations of 3D planning are related to higher required costs and time in acquiring images as well as increased radiation exposure, and therefore its applications are limited to more complex cases [52].

Operator dependence is one of the principal characteristics that affects accuracy in conventional digital templating [53], as well time consumption and workload requirements that represent issues that are debated in the literature; therefore, AI is a promising tool for assisting surgeon in performing preoperative planning, aiming at improvement in its efficacy and accuracy [54]. The promised actions include automatic identification of anatomical landmarks, and suggestion about optimal THA implant component positioning and sizing, limit operator-related influences and time consumption [54]. The literature about this topic has been growing in recent years, thanks to the potential benefit on preoperative surgical planning. Several ML models have been developed and tested, and performances of these algorithms have been evaluated with promising results.

Zampogna et al. [55] analysed six supervised ML algorithms trained for 2D digital preoperative templating of 800 patients undergoing to primary posterolateral THA. The authors conducted a retrospective analysis with the aim of evaluating the accuracy of various supervised ML (sML) models in predicting the reliability of preoperative planning in THA. Sex, age, biometric measures, comorbidity burden, perioperative blood test, length of stay after surgery, and ASA score were integrated with data from standard pelvic radiographs. The accuracy of each sML, defined as the concordance between the predicted model and size of the component by the algorithm and the implanted one, was established, and specific type of ML model called KNN resulted as the most accurate ML model (98%) for preoperative planning for both acetabular and stem positioning.

The application of ML algorithms was studied in 3D digital templating. AI-HIP [56] is an example of DL-based system developed to assist surgeons in preoperative templating on CT images. The system was trained to identify anatomical landmarks on preoperative CT images, including eventual deformities, and to perform postoperative simulations comprehensive of planned implant size, positioning and predicted postoperative offset, leg-length, and coverage. Chen et al. [56] conducted a validation study of the system, feeding AI-HIP with 3000 CT images, and evaluating its performance on 60 cases, which were compared with 60 conventional X-rays-based THA templating; they showed better accuracy using AI-HIP templating compared with X-ray-based planning for both cup and stem size. Functional postoperative parameters showed no statistically significant differences between the two groups collected preoperatively and 12 weeks postoperatively. In the study conducted by Huo et al. [57], AI-HIP-based templating was compared with 2D digital planning and 3D CT-based templating. Fifty-nine patients undergoing primary uncemented THA were evaluated, and predicted component size and positioning and time spent in templating were recorded and compared. The authors found similar results in accuracy for AI-HIP templating and 3D planning, but templating time was significantly lower for AI systems.

AI integration in preoperative templating promises several innovations that would reduce time consumption and workload; however, these could standardize an operator-dependent process. AI offers a more time-saving process, capable to implement visualization in a 3D manner. Moreover, immersive and processed images could be obtained, the positional deviation could be corrected, and postoperative simulation could be performed. Finally, AI systems could be useful for educational purposes, supporting practicing surgeons in acquiring templating skills [57]. Being a field under development, the implementation of AI systems in preoperative planning for primary THA presents some limitations: ML models are a data-dependent technology, and restricted sample size may limit its ability. Better performances could be reached with heterogeneous data, gathered from several institutes and performed under diverse conditions. Most of the analysed studies were from single institutions, determining a bias also with the learning curve of the algorithm because of image acquisition methods specific to the individual centre, variations in the used equipment, and techniques employed by the operators. For this reason, most authors recommend conducting subsequent analyses with a larger, multi-centre samples to enhance the generalizability of the findings and to mitigate potential biases.

## 6. Postoperative Evaluations and Detection of Complication

Second-generation uncemented implants registered successful results at 20 years in terms of survival [58]. Thanks to these excellent results, the burden of THA procedures is progressively increasing [59]; at the same time, early recognition of THA implant failure is under the attention of clinicians [60]; therefore, authors studied AI potentialities in independent screening of radiographic follow-up to detect failing implants.

As supported by recent consensus, CRP is used as first-line serum marker in suspected periprosthetic joint infection (PJI) [61]. Others serum markers, such as WBC count, should not be considered alone due to the low sensitivity value reported [62,63].

Loppini et al. [64] performed a retrospective analysis with the aim to develop a DL algorithm able to automatically detect implant loosening from follow-up plain radiographs from patients operated of revision THA for implant failure. Preoperative revision X-rays diagnosed for stem loosening, acetabular cup loosening, malpositioning of the implant, polyethylene wearing, or periprosthetic infection were reviewed. Radiological signs for each condition were incorporated into the algorithm and DL net was tested and validated for the classification of preoperative X-rays with signs of failure. The examined algorithm achieved very high accuracy (>97%) in detecting the incipient radiological signs of implant failure, supporting the application of the AI system in clinical practice.

In the prevision of THA revision surgery, implant identification is required to plan surgical instrumentations to use during surgery and to minimize the need for total implant revision. Conventionally, the identification is reached by consultation of medical records, technical galleries, and recall of implant labels. This process may become extremely arduous, expensive, and time consuming, and it is associated with failure in patients with older implants, operated in facilities abroad, and when it is difficult to reach the original medical record [65]. AI, thanks to classifying abilities and high data handling, may support surgeons in this process. Bojani et al. [66] tested a Deep Convolutional Neural Network (DCNN) in recognizing implant types and trademarks of THA implants before revision surgery. Authors developed a Supervised Learning system for recognition of known implants, reporting excellent results in terms of accuracy (100%), and demonstrating how this technology could significantly impact revision surgery templating and reduce time and costs required for implant recognition. Several AI systems were assessed in recent years. The THA-AID DL tool was trained by more than 240,000 plain radiographs for the identification of 20 femoral stems and 8 acetabular cups in AP, lateral, and oblique X-rays; it registered a high accuracy for both implant components (98.8%) [67]. The model could serve as a decision aid tool and be included in the preoperative assessment in revision THA surgery.

Periprosthetic joint infections (PJI) represent another challenging complication of THA procedures. After the increasing burden of THA performance, PJI are projected to increase, being among the most common causes of revision surgery. The lack of a defined diagnostic scheme and the multiple criteria that contribute to PJI diagnosis according to 2018 ICM definition, raise the complexity in defining patient at risk; a clear diagnosis could be reached only after surgical debridement procedure with collection of sample for histological and microbiological analysis [68].

Machine Learning was tested with the aim to assist in assessment of PJI, aiding the definition of a scoring system, and the detection of patients at risk. Most applications of ML in this field regard the development of algorithms as a help in determining the probability of PJI, given a set of data. For instance, ML was tested to develop a PJI probability algorithm from data collected by synovial fluid (SF) tests [69,70]. Paranjape et al. [69] tested the probability of PJI from a set of biomarkers from SF in a patient treated with total joint replacement, including SF-C Reactive Protein (SF-CRP), Alpha defensin (AD), white blood count (WBC), and a pattern of microbial antigen panel. Unsupervised ML was employed to define clusters of cases from data analysis performed by the machine; then, a decision tree algorithm was defined and provided a probability risk of PJI instead of a binary outcome. Wu et al. [70] studied an AI algorithm to determine surgical site infections (SSI) in patients with total hip or knee arthroplasty. SSI were defined as infection occurring at the site of surgery [71,72]. The authors built up a DL model tested in multi-centre retrospective analysis to perform regression and clusterisation of dataset using a decision tree scheme, to evaluate the risk of SSI from data collected by electronical medical reports (EMRs), including medical and nursing notes. The tested net demonstrated promising results; interestingly, the analysis of clinical notes by NLP technologies revealed an added benefit that contributed to enhance the model performances. This tool indeed was capable to analyse unstructured data including clinical descriptions and observation (such as wound descriptions, clinical conditions, etc.) that were barely considered conventionally. Di Matteo et al. [73] demonstrated that ML models could archive good performances in prediction of PJI in patients undergoing revision THA, strongest predictors included serum C-reactive protein, systemic markers of inflammation, and serum red blood cell distribution. Even more development and validation of AI models are needed for proper clinical application, and these studies evidence that AI tools could be impactful in the future management of PJI.

## 7. Limitations and Concerns

Even if the subject of AI is gaining popularity in healthcare, its application in health systems is not without limitations and concerns. Most of the cited works complaint limitations regarding commons issues, and datasets collected by single institutions, with poor validity in other settings. Studies of AI-aided image analysis underlined the doubtful validity of AI systems tested in other conditions, affecting the external reproducibility of results; therefore, the reliability in the spread of clinical settings could be reduced [44,45]. Being a data-dependent technology, ML-analysed systems increase validity if more numerous and diverse data are provided, and most manuscripts suggest further investigations to assess the outer performance of the tested ML model.

Another issue is represented by the so-called “black-box AI” which, particularly in deep nets, refers to the impossibility to trace the processes performed by the machine underneath a certain decision or output [11,30]. This feature, intrinsic to ML models, could be remarkable in external validity of models developed and tested in clinical settings; however, none of the studies analysed so far seem to account for this issue.

The issue of “alignment” encompasses several critical facets. Firstly, there is the challenge of data alignment, ensuring that the vast datasets used to train AI algorithms are representative, unbiased, and accurately reflect the diverse patient populations undergoing hip surgery. Biases in training data can lead to algorithms that perform sub-optimally or inequitably across different demographic groups. Secondly, clinical alignment is crucial. The outputs and recommendations generated by AI systems must align with established surgical best practices, the nuanced clinical judgment of experienced surgeons, and the specific needs of individual patients. Over-reliance on AI without critical oversight could lead to deviations from optimal surgical techniques. Finally, ethical alignment presents a complex hurdle. Ensuring that AI applications in hip surgery adhere to ethical principles of patient autonomy, data privacy, and accountability is paramount. Misaligned AI systems could potentially erode patient trust or introduce unintended consequences, highlighting the necessity for careful development and rigorous validation in this sensitive medical domain.

Academic reports that claim about the added value of AI aided healthcare are increasing. Most of the presented work was based on the same rational, i.e., comparative performance of a developed AI system and a group of clinicians with different level of experience in performing the same task. All the reports exposed with enthusiasm the results achieved by AI, especially the superiority in time preservation and performance comparable with a specialist with high expertise. Some scholars affirm that AI performance in medicine is overestimated and that it could lead to misinformation regarding the actual potentialities this technology [74,75]. The lack of contextualization and transparency in reporting AI outperformances harnesses the clarity of the studies and preclude the fully understanding of novelty carried by these tools.

Cabitza et al. [30] critically examines the unintended consequences of Machine Learning (ML) in medicine, highlighting risks such as bias, lack of transparency, and over-reliance on automated decisions. While ML enhances diagnostics and predictive modelling, its “black box” nature raises ethical concerns regarding accountability and interpretability. Moreover, excessive trust in AI may reduce clinical oversight, leading to an increase in errors and risk of malpractice. Only rigorous validation, ethical oversight, and clinician–AI collaboration and supervision may ensure ML safety and effective integration of AI into medical practice.

## 8. Conclusions

Artificial Intelligence (AI) is increasingly demonstrating its potential in orthopaedics, particularly in fracture diagnostics, preoperative planning for hip surgery, implant recognition for revision procedures, and predictive modelling of periprosthetic infections and aseptic complications. Our review of current of the literature highlights promising advancements in these applications, suggesting that AI-driven tools can significantly enhance clinical efficiency and decision-making in orthopaedic surgery.

In femoral fracture and secondary OA, AI-powered algorithms, particularly DL models, showed high accuracy in image-based assessments. These tools offer a significant advantage, reducing time spent in diagnostics, with good inter-observer variability and reliance with expert radiologists; these tools may be particularly beneficial in emergency settings. Similarly, AI applications in preoperative planning and templating for primary and revision THA surgery may facilitate surgical decision-making, providing precise anatomical measurements and implant selection guidance, contributing to the work of the surgeon to develop patient-specific surgical strategies. Such advancements contribute to the optimization of surgical workflows and may potentially improve clinical outcomes.

Beyond diagnostics and planning, AI-based predictive analytics is emerging as a valuable tool for assessing the risk of periprosthetic infections and aseptic complications. ML models can analyse large datasets to identify patient-specific risk factors, associated intraoperative variables, and postoperative indicators that may predispose patients to complications. Such predictive capabilities could promote early intervention strategies, personalized patient management, and improve postoperative surveillance.

While the benefits of AI in orthopaedics are evident, reducing time, costs, and clinical workload while enhancing diagnostic accuracy and surgical planning, certain challenges remain. One limitation concerns the trustworthiness of AI models, because studies are conducted in controlled environments with high-quality datasets, limiting real-world applicability. Additionally, the hype surrounding AI may create unrealistic expectations, potentially leading to a premature unsupervised clinical adoption without sufficient validation. Further research, including prospective clinical trials and real-world implementation studies, is necessary to confirm AI’s reliability, safety, and long-term impact in daily orthopaedic practice.

In conclusion, AI presents a transformative opportunity in orthopaedics, particularly in fracture diagnostics, preoperative planning, implant recognition, and risk prediction of surgical complications. It could be expected that in the near future, AI technologies will be implemented to improve intraoperative precision of surgical implants positioning, with the aim to optimize outcomes. While current evidence underscores its clinical value and operational efficiency, further validation and integration into standardized clinical workflows are essential to maximize its accuracy, applicability, and ethical deployment in orthopaedic surgery.

## Figures and Tables

**Table 1:** Features and issues related to different kinds of Machine Learning (ML).

Features	Supervised ML	Unsupervised ML	Reinforcement Learning
Definition	Learning model processing labelled data with input and output well specified	Learning model working with unlabelled data	Learning model developing from interaction with environment
Data	Labelled data	Unlabelled data	Action–reward-based data
Aim	Classify and predict output on new data on the basis of trained models	Find out models or structures of data	Searching for a cumulative reward
Issues to solve	Classification regression	Clustering	Resources managing, controls.
Supervision	Complete supervision	No explicit known supervision	Environment feedback
Feedback	No known feedback	Correct labels	Rewards and penalty

**Table 2:** Comparison of functionalities and application of Machine Learning and Deep Learning. ML Machine Learning; DL Deep Learning.

	Machine Learning	Deep Learning
**Definition**	ML uses learning techniques (Supervised Learning, Unsupervised Learning) to predict model functioning by the data provided.	DL uses neural network to interpret unprocessed databased.
**How it works**	Algorithms usually require human examination to evaluate dataset variables and features.	DL does not require human intervention.
**Database**	ML could run with small datasets.	DL requires large databases.
**Application in orthopaedics and clinical research**	Predicting risk of complications or revision surgery; classifying orthopaedic conditions based on clinical or radiographic data; decision-support tools using arthroplasty registries.	Automated detection of fractures or degenerative changes on X-ray, CT, MRI; image segmentation for surgical planning; multimodal prognostic modelling combining imaging and clinical variables.

## Data Availability

No new data were created or analyzed in this study.
